# Time Course of Physiological and Psychological Responses in Humans during a 20-Day Severe-Cold–Acclimation Programme

**DOI:** 10.1371/journal.pone.0094698

**Published:** 2014-04-10

**Authors:** Marius Brazaitis, Nerijus Eimantas, Laura Daniuseviciute, Neringa Baranauskiene, Erika Skrodeniene, Albertas Skurvydas

**Affiliations:** 1 Sports Science and Innovation Institute, Lithuanian Sports University, Kaunas, Lithuania; 2 Department of Educational Studies, Kaunas University of Technology, Kaunas, Lithuania; 3 Department of Laboratory Medicines, Medical Academy, Lithuanian University of Health Science, Kaunas, Lithuania; University of Sao Paulo, Brazil

## Abstract

The time course of physiological and psychological markers during cold acclimation (CA) was explored. The experiment included 17 controlled (i.e., until the rectal temperature reached 35.5°C or 170 min had elapsed; for the CA-17 session, the subjects (*n* = 14) were immersed in water for the same amount of time as that used in the CA-1 session) head-out water immersions at a temperature of 14°C over 20 days. The data obtained in this study suggest that the subjects exhibited a thermoregulatory shift from peripheral-to-central to solely central input thermoregulation, as well as from shivering to non-shivering thermogenesis throughout the CA. In the first six CA sessions, a hypothermic type of acclimation was found; further CA (CA-7 to CA-16) led to a transitional shift to a hypothermic–insulative type of acclimation. Interestingly, when the subjects were immersed in water for the same time as that used in the CA-1 session (CA-17), the CA led to a hypothermic type of acclimation. The presence of a metabolic type of thermogenesis was evident only under thermoneutral conditions. Cold-water immersion decreased the concentration of cold-stress markers, reduced the activity of the innate immune system, suppressed specific immunity to a lesser degree and yielded less discomfort and cold sensation. We found a negative correlation between body mass index and Δ metabolic heat production before and after CA.

## Introduction

Optimal thermoregulatory functioning, which is affected by adaptation to chronic cold exposure, is of critical importance for the survival, health and well-being of humans who are exposed to occupational and/or recreational extreme-cold conditions. It has been well demonstrated that successful whole-body adaptation to cold in humans can be expressed differently, as indicated by the development of the metabolic (as evidenced by no change in rectal temperature (T_re_), whereas skin temperature (T_sk_) and metabolic heat production (MHP) increases), hypothermic (as evidenced by no change in T_sk_, whereas T_re_ and MHP decreases) or insulative (as evidenced by no change in T_re_ and MHP, whereas T_sk_ decreases) type of acclimation (induced experimentally) or acclimatization (induced naturally) [19], [56], [4], [53], [47], [24], [29]. The existence of insulative–hypothermic [41], [56], [24], [29] or metabolic–insulative [4], [29] subtypes is sometimes evident as well. Nevertheless, it has been shown that all three possible types of cold adaptation were present in winter swimmers [27], [53] and female pearl divers (AMA) from Korea [21]. Both groups adapted to acute cold by swimming or diving outdoors regularly during winter for several years; in those conditions, the water temperature decreases down to 2°C or 10°C, respectively. It has been suggested that such level of cold adaptation might be sufficient to enhance most of the physiological responses involved in exposure to cold in humans [27]. These differences in the types of adaptation observed to date have been explained by the different experimental conditions used in individual experiments, and by the different physiological states of the subjects [5], [16], [22], [29].

The origin of thermoregulation in non-cold-adapted humans involves metabolic, hypothermic and insulative patterns of physiological responses to exposure to acute cold (evidently, the T_sk_ decreases to a greater extent that does the T_re_, whereas MHP increases). It appears that adaptation to repeated cold exposure is mainly expressed by a proportional change in the magnitude of the physiological response of the three types of acclimation described above. More than four decades ago, Skreslet, Aarefjord [44] suggested that the different types of cold acclimation (CA) do not represent mutually exclusive physiological states; rather, they are different successive stages of the development of complete cold adaptation. Based on data obtained from three male scuba divers, those authors hypothesized that CA develops progressively, beginning with a response to cold with the characteristics of the metabolic type, proceeding through an intermediate phase during which cold responses are of the hypothermic type, and resulting in the development of the insulative type of acclimatization. Since then, however, despite the large number of studies performed on this subject, this hypothesis has not been tested properly. In most studies, the researchers studied only the pre- and post-effects of CA. However, to test this hypothesis, a day-to-day evaluation of at least three generally accepted physiological markers (T_sk_, T_re_ and MHP) throughout whole-body CA in cold water is necessary.

Acute cold-stress exposure impairs vigilance, overall mood [28] and motor and cognitive performance [12]–[13], [28], [6], [38], [7]. The hypothalamic–pituitary–adrenal axis, autonomic nervous system and immune system are sensitive to stress [33]. The commonly used biomarkers of stress are cortisol and catecholamine levels [33]. When activated by cold stress, the adrenal medulla secretes catecholamine epinephrine [25], [15], [39] and norepinephrine [56], [34], [51] into the blood plasma. Epinephrine increases heat production in skeletal muscles and white adipose tissue [2]–[3], [42]–[43], whereas exogenous norepinephrine activates cutaneous vasoconstriction [56] and thermogenesis in brown adipose tissue (BAT) [10]. Cortisol can weaken the activity of the immune system [36], [20]. The immune function seems to be stimulated by acute stress, but is suppressed by chronic stress [14], [55], [40], [26]. This dual response can be beneficial for some types of immune responses, but deleterious for other types. There is no current consensus regarding the response of the immune system, stress biomarkers and psychological perception to severe chronic cold stress.

The main purpose of our investigation was to determinate whether 17 head-out acute cold-water immersions over 20 days is sufficient to develop CA by remodelling proportional changes in the development of metabolic versus hypothermic versus insulative patterns of physiological response. It has been postulated that thermal acclimatization reflects a transition from one steady state to another [23]. It can be hypothesized that the day-to-day time course of physiological responses during CA is expressed as a transitional shift from shivering to non-shivering thermogenesis, from the development of metabolic to hypothermic to insulative pattern of acclimation and from peripheral-to-central to central temperature input thermoregulation. We also expect that chronic cold stress will yield a greater activation of the specific, rather than the innate, immune system. Moreover, a greater amount of white adipose tissue (as indicated by body mass index (BMI) and subcutaneous fat thickness) in the subjects implies a lesser (more blunted) increase in non-shivering thermogenesis [52], [35] after CA.

## Materials and Methods

### Participants

Fourteen healthy males with no history of cold and/or heat injury were recruited into this study. The subjects were moderately physically active (<2 h·week^−1^), but did not participate in any formal physical exercise or sports program. They had not been involved in any temperature-manipulation programme or procedure for at least 1 year. Each subject volunteered to participate in the study after being informed of the purpose, experimental procedures and known risks of the study. Each subject read and signed a written informed consent form consistent with the principles outlined in the Declaration of Helsinki. A signed permission-to-participate form from an independent medical doctor was also required from each subject before the study. The Kaunas Regional Research Ethics Committee approved this study (2011-10-12 No. BE-2-41).

### Preliminary measurements

Anthropometrical characteristics of subjects are presented in Table 1. Subjects' weight (kg), body fat (%), body mass index (TBF-300, Tanita, UK Ltd. Philpots Close, UK) and height (cm) were measured. Body surface area (m^2^) was estimated by 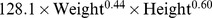 [49]. Skinfold thickness (mm) was measured (Skinfold caliper SH5020, Saehan, Masan, Korea) at 10 sites: chin, subscapular, chest, side, suprailium, abdomen, triceps, thigh, knee, and calf [31] and mean subcutaneous fat thickness was calculated [1].

**Table 1:** Anthropometrical characteristics of subjects.

Age, yr	21.6±0.5
Height, cm	181.9±1.7
Mass, kg	79.3±1.9
Body surface area, m^2^	2.00±0.03
Mean subcutaneous fat, mm	9.0±0.6
Body fat, %	19.2±1.5
Body mass index, kg/m^2^	20.7±1.3

Values are means ± SEM.

### Body temperature measurements

Participants' T_re_ was measured throughout each CA session by a thermocouple (Rectal Probe; Ellab, Hvidovre, Denmark; accuracy ±0.01°C) inserted to a depth of 12 cm past the anal sphincter. The rectal thermistor sensor was placed by each subject. T_sk_ was measured (DM852; Ellab; accuracy ±0.01°C) before and at the end of the water-immersion session in all CA sessions, with thermistors taped at three sites: the back, thigh and forearm. The mean T_sk_ was calculated using the Burton [9] equation: 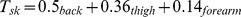. Muscle temperature (T_mu_) was measured before and at the end of the water-immersion session on CA-1, CA-16 and CA-17 days. Intramuscular temperature was measured with a needle microprobe (MKA; Ellab) inserted at a depth of 3 cm under the skin covering the gastrocnemius lateral muscle of the right leg. At the insertion point, calf skin temperature was also measured, only to calculate the temperature gradient between T_mu_ and calf T_sk_. Skin preparation before each intramuscular temperature measurement involved skin shaving and disinfection (before and after insertion of the microprobe) using a cotton/wool tampon soaked with medicinal alcohol. No local anesthesia was administered prior to insertion. After the first measurement, the insertion area was marked with a circle with a diameter of 0.5 cm to ensure repeatability and use of the same insertion point between measurements (pre- and post-session; different session days: CA-1, CA-16 and CA-17).

### Blood variables

Blood samples were collected by venipuncture into vacuum tubes (EDTA-K3, 3 ml) before and at the end of water immersion session on CA-1, CA-16, and CA-17 days, mixed gently by inverting 8–10 times, and kept at room temperature until analysed for differential blood cell counting of neutrophils, leucocytes, lymphocytes and monocytes. Blood samples were analysed 1 to 2 h after blood collection using an automated haematology analyser, XE-5000 (Sysmex Corp., Kobe, Japan).

Blood samples for measurement of epinephrine and norepinephrine concentrations were collected in vacuum tubes using EDTA as an anticoagulant (EDTA-K3, 3 ml), mixed gently by inverting 8–10 times and kept at 2–8°C until centrifugation. Blood samples were centrifuged at 1200×*g* for 15 min within 30 min of blood collection. Plasma samples were separated as soon as possible (maximum 10–15 min) from the red cells after centrifugation, and kept at −70°C until analysis. Epinephrine and norepinephrine concentrations were measured using an ELISA kit (Gemini analyser, Stratec Biomedical GmbH, Birkenfeld, Germany).

Blood samples for measurement of cortisol concentration were collected by venipuncture into vacuum tubes for serum separation using a gel separator (5 ml). Blood samples were allowed to clot, and the serum was separated by centrifugation at 1200×*g* for 15 min. The serum samples were aliquoted and stored at −70°C until analysis. Concentration of cortisol was measured using an automated enzyme immunoassay analyser, AIA-2000 (Tosoh Corp., Tokyo, Japan).

### Spirometry and cardiovascular responses

Heart rate (HR) was measured throughout each CA session with a heart rate monitor (S-625X, Polar Electro, Kempele, Finland). Resting, end and mean HR value during immersion (measured every 5 min) sessions were registered.

Mobile spirometry system (Oxycon Mobile, Jaeger/VIASYS Healthcare, Hoechberg, Germany) was used to measure pulmonary gas exchange (i.e. at rest and water immersion procedure). This system employs a tightly fitting facemask covering nose and mouth with a lightweight integrated flowmeter (Triple V volume sensor; 45 g) with a dead space of 30 ml. It monitors ventilatory parameters, oxygen uptake and VCO_2_ production on a breath-by-breath basis. The processing, recording and battery system is comprised of two units attached to a belt, which during the testing was hungered as close as possible to the subjects' noise and mouth area. Data were stored on memory card and PC hardware. Calibration of this instrument was performed prior to recording according to the manufacturer's manual employing the automatic volume- and gas-calibration functions. A flow-volume sensor calibration procedure assures that the Oxycom quantification system (including the amplifier, Triple V sensors, and pressure transducer) is functioning correctly. The gas analyser and delay time calibration was also automatic, as provided by the manufacturer: a calibration gas at 180 kPa (15.2% O_2_, 5.02% CO_2_ and 79.62% N_2_) was introduced to the Oxycon to attain gain, offset and delay times within 1%. Oxygen consumption was recorded in 5-s increments. Data collected during each first 5 min of 20 min immersion were not used in any related calculations, as recommended [48], because of reflex hyperventilation caused by cold water immersion. Because water immersion time during session differed between subjects mean value per session of VO_2_ (in ml/min/kg; l/min) and VCO_2_ (l/min) was calculated.

Metabolic heat production (MHP) (in Watts) was calculated from the respiratory gas exchange measurements of VO_2_ (in l/min) and the respiratory exchange ratio (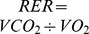) according to Peronnet, Massicotte [37]: 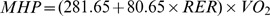.

The cold shock response during the first minute of cold water immersion was determined by measuring reflex hyperventilation (in V′E L/min) [16]–[17], [30] throughout CA sessions.

### Perceptual measurements

The method to measure subjective ratings for the whole-body has been described elsewhere [18], [8]. Briefly, ratings of thermal sensation ranged from 1 (very cold) to 9 (very hot), with 5 being neutral. The shivering sensation ranged from 1 (vigorously shivering) to 4 (not at all) being neutral. The rating of perception was reported by the participants every 5 minutes during passive cooling exposure in all experiments. Mean rating score value per session were calculated.

### Cold strain index (CSI)

A CSI based on core (T_re_) and mean skin temperatures (T_sk_) and rates cold strain on a universal scale of 0–10: 1–2 (no/little cold strain); 3–4 (low cold strain); 5–6 (moderate cold strain); 7–8 (high cold strain); and 9–10 (very high cold strain), and is as follows [32]: 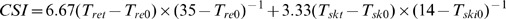.

The measurements for CSI were taken before (T_re0_, T_sk0_) and at the end of passive cooling (T_re*t*_, T_sk*t*_), on CA-1, CA-16 and CA-17 days; 14°C – water temperature; 35°C – T_re_ threshold. T_re_ and T_sk_ were assigned with weight by using a constant of 6.67 and 3.33, respectively.

### Rationale for experiment and research design

**Figure 1:**
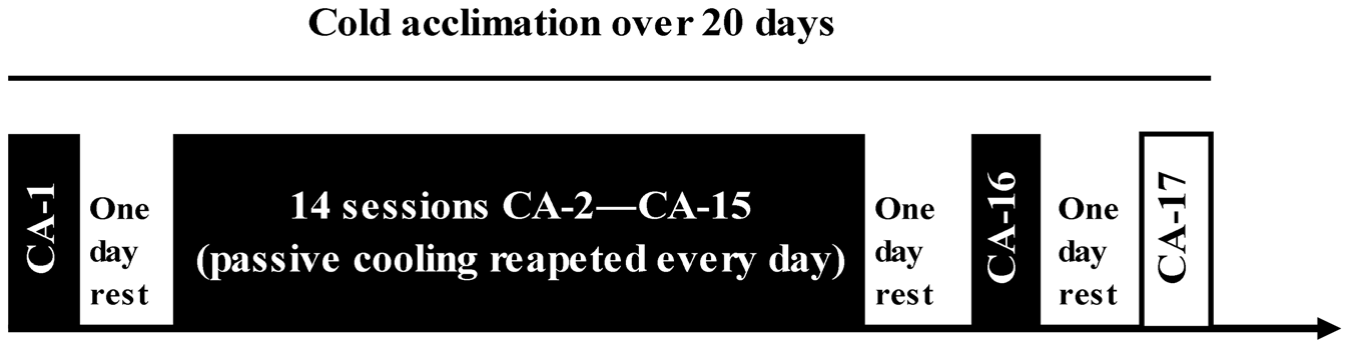
Research design. Cold acclimation (CA) protocol–17 head-out cold-water immersions (CA-1 to CA-17) over 20 days.

The CA experiment was designed to induce whole-body CA and explore the effect of CA on the time course of the physiological and psychological markers measured. It involved 17 controlled (until the T_re_ reached 35.5°C or 170 min had elapsed; for the CA-17 session, the subjects were immersed in water for the same amount of time as that used in the CA-1 session) head-out water immersions at a temperature of 14°C over 20 days. The research design is presented in Figure 1. Subjects were required to come to the laboratory on 19 separate occasions. The initial visit involved familiarization with the experimental procedures and equipment, and the preliminary measurements were sampled (see “Preliminary measurements”). At least 2 days later, the participants started the protocol of 17 CA sessions over 20 days (see “CA protocol”).

### CA protocol

The CA protocol comprised 17 sessions of head-out cooling over 20 days. One day of rest was applied between the CA-1 and CA-2, the CA-15 and CA-16 and the CA-16 and CA-17 sessions. Testing was conducted from August to December, to limit the occurrence of CA through casual exposure to low ambient temperatures (the ambient temperature during the testing period ranged from 5 to 21°C at day-time and from 0 to 14°C at night-time (these data were obtained from a local weather service)). The experiment was conducted indoors at the same time of day (from 7:00 a.m. to 11:00 a.m.). The subjects refrained from consuming any food for at least 12 h before the experiment. To standardize the state of hydration and the feeling of thirst, subjects were allowed to drink still water as desired until 60 min before the water-immersion session. The experiments were performed at a room temperature of 22°C and a relative humidity of 60%. Prior to each CA immersion, the subjects rested in a semi-recumbent posture for 10–15 min dressed in a T-shirt, swim shorts and socks. The resting pulmonary gas exchange in the subsequent 20 min was recorded in the same semi-recumbent posture (the resting pulmonary gas exchange was measured only in the CA-1, CA-16 and CA-17 sessions). T_sk_, T_mu_, T_re_ and HR stabilization were assessed, the control measurements of T_sk_, T_mu_, T_re_ and HR were performed and blood samples were collected from a vein and stored for later analyses (the T_mu_ was measured and blood samples were taken only in the CA-1, CA-16 and CA-17 sessions). Subsequently, volunteers were prepared for the water-immersion procedure and the cooling protocol began. During cooling, the subjects were asked every 20 min to step out of the bath and rest for 10 min in the room environment, and then to return to the water bath for the next 20 min of immersion. The temperature of the water bath was 14°C and a head-out water immersion procedure was used. In the first experimental condition (i.e., from the CA-1 to the CA-16 sessions), this head-out water immersion procedure continued until the T_re_ had decreased to 35.5°C or until 170 min of the T_re_ had elapsed in total (120 min maximum of total immersion time), at which time the immersion ended. The exposure time until the T_re_ was achieved was recorded. In the second experimental condition (in the CA-17 session), the subjects were immersed in water for the same time as that used in the CA-1 session and the end T_re_ was sampled. During immersion and resting, the subjects remained in the semi-recumbent posture with their arms folded across their chests and their legs almost straight and together. HR, T_re_ and rating of perception were recorded every 5 min throughout the cooling procedure. Pulmonary gas exchange in all 17 CA sessions was recorded only during water-immersion time (every 20 min of immersion). Immediately after this procedure, the volunteers were towel dried, T_sk_ and T_mu_ measurements were repeated and blood samples were taken. During the experimental period, no exercise was allowed.

### Statistical analysis

The data were tested for normal distribution using the Kolmogorov–Smirnov test: all data were normally distributed. The effect of CA on dependent variables (body temperature, cooling time, pulmonary gas exchange, CSI, HR and blood samples) was analyzed via one-way analysis of variance for repeated measures (pre- *vs.* post-cooling; between different CA sessions). The non-parametric Wilcoxon signed-rank test was used to compare the changes in subjective ratings of perceptions (thermal, comfort and shivering sensations) between CA sessions. Pearson's correlation coefficients were used to identify relationships between variables. Data are reported as means ± standard error of measurement (SEM) and the level of statistical significance of all tests was set at *P*<0.05. Statistical power (SP) was calculated for all mechanical indicators based on an alpha level of 0.05, sample size (*n* = 14), standard deviations and average level of variables.

## Results

### The effect of CA on body temperature and duration of immersion

**Figure 2:**
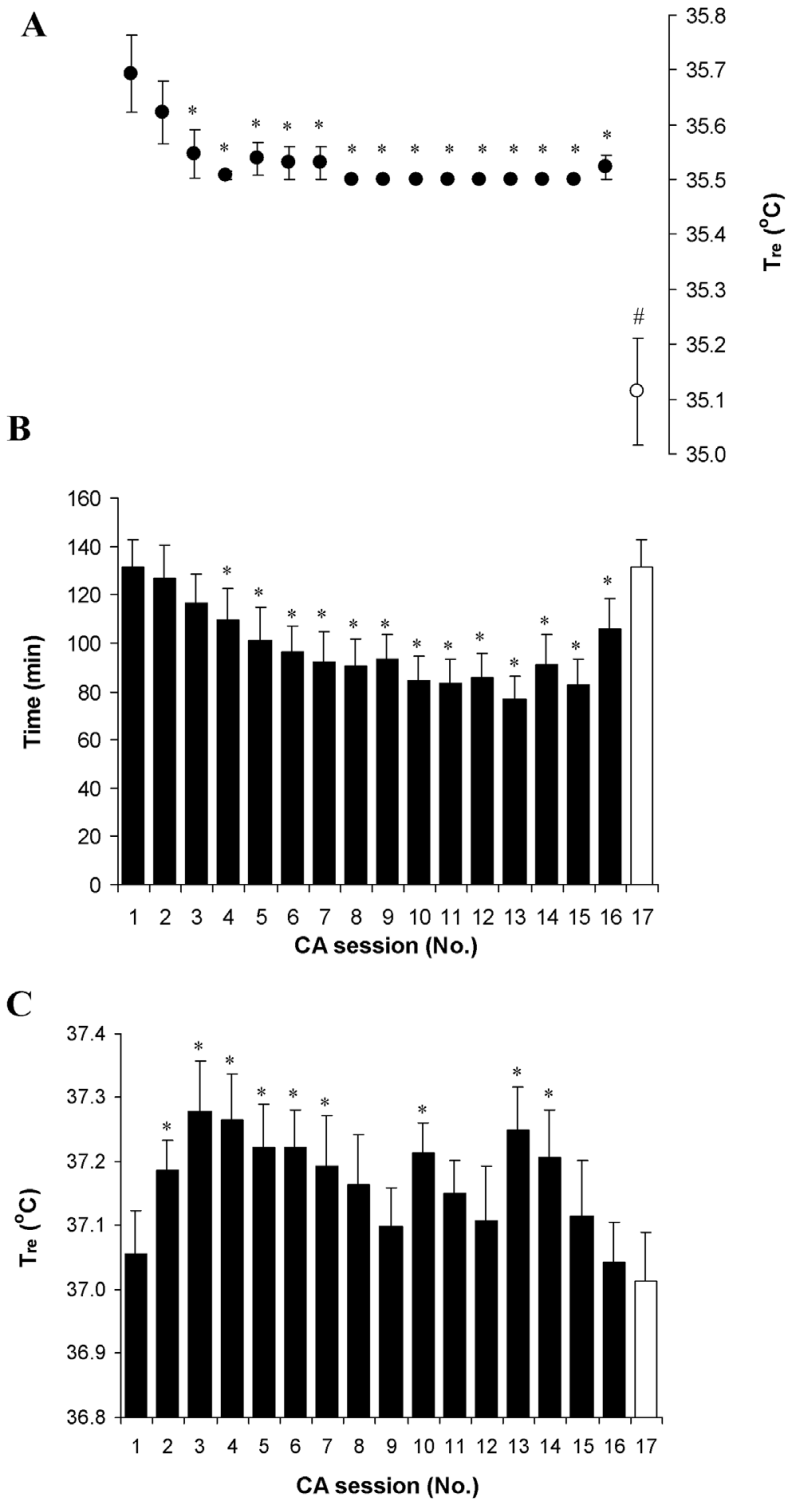
Cooling time and rectal temperature throughout the cold acclimation. Cooling time (B) and rectal temperature before (C) and after (A) cooling. * *P*<0.05, compared with CA-1; # *P*<0.05, compared with CA-17. Values are means ± SEM.

**Figure 3:**
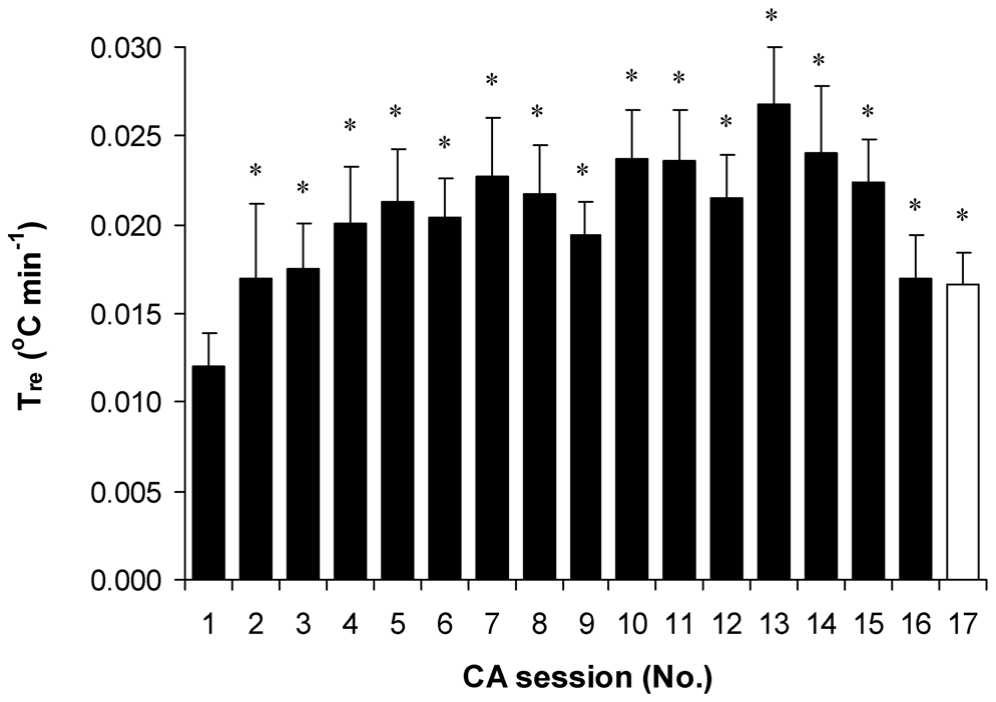
Rate of decrease in rectal temperature during cooling throughout cold acclimation. * *P*<0.05, compared with CA-1. Values are means ± SEM.

**Figure 4:**
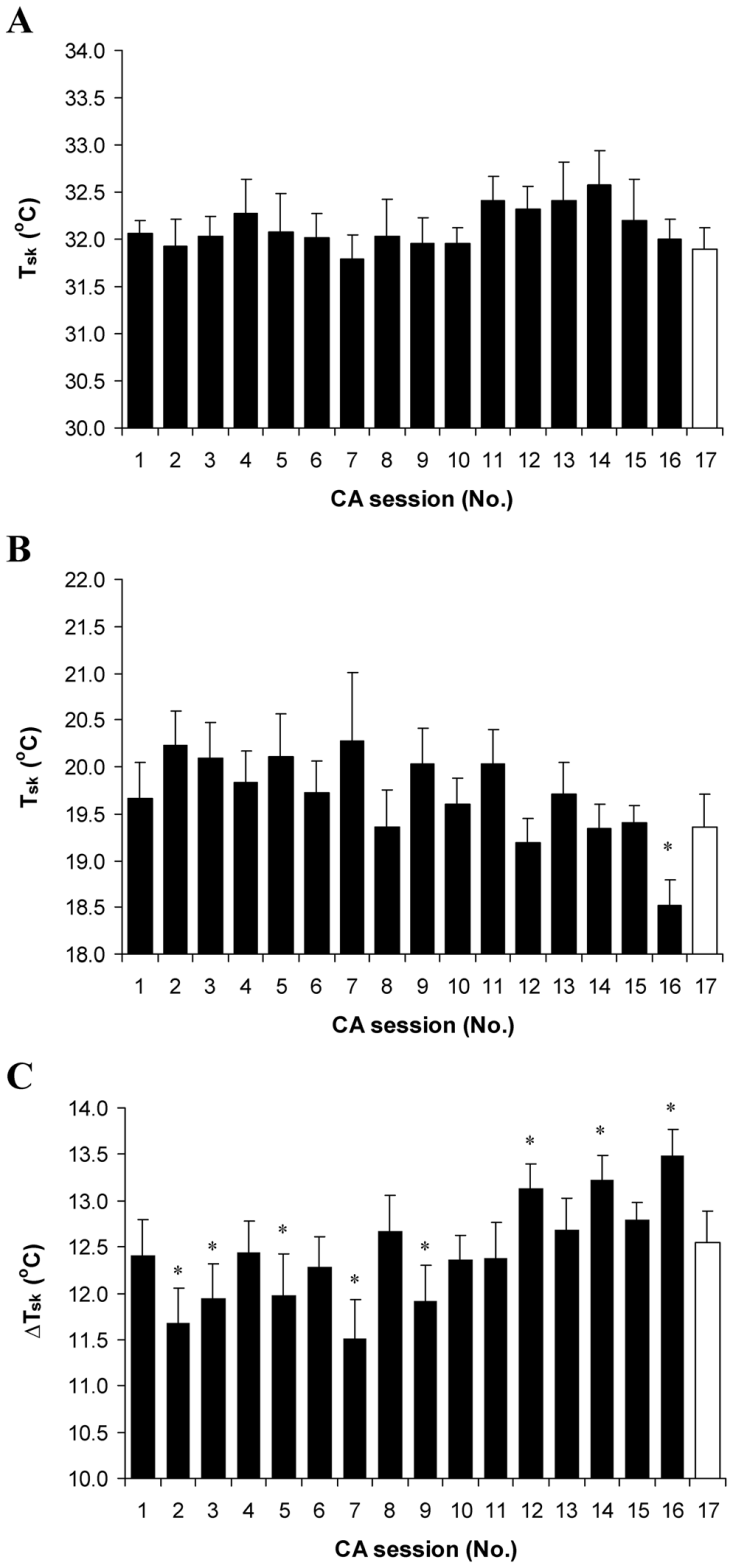
Skin temperature throughout cold acclimation. Skin temperature before (A) and after (B) body cooling, and change from before to after body cooling (C). * *P*<0.05, compared with the CA-1. Δ T_sk_  =  mean difference between T_sk_ before and T_sk_ after cold exposure. Values are means ± SEM.

The head-out cooling protocol significantly reduced T_re_, T_mu_ and T_sk_ in all subjects (*P*<0.001; SP>99%; comparison of pre- with post-cooling). Evidently, the set point of T_re_ 35.5°C was reached in all subjects in the CA-4 session (Figure 2A). Cooling time in cold water until a T_re_ of 35.5°C was reduced consistently throughout the CA protocol, and was significantly shorter from the CA-4 to CA-16 sessions compared with CA-1 (*P*<0.001; Figure 2B). CA resulted after an approximately 0.6°C greater decrease in T_re_ when the subjects were immersed in water for the same time as that used in CA-1 (*P*<0.001; SP>95%; 1.33±0.12°C and 1.9±0.14°C in CA-1 and CA-17, respectively). The rate of decrease in T_re_ increased consistently up to the CA-13 session (*P*<0.001; SP>80%; 0.014±0.003°C·min^−1^ and 0.027±0.003°C·min^−1^ in CA-1 and CA-13, respectively), and then decreased, but did not reach the pre-acclimation level (*P*<0.0.5; SP>45%; comparison of CA-1 with CA-16 and CA-17; Figure 3). A significantly greater decrease in T_sk_ after body cooling was found only in CA-16 (*P*<0.05; SP>45%; 19.0±0.4 and 18.3±0.3 in CA-1 and CA-16, respectively) (Figure 4B, Table 2). The change in T_mu_ between before and after body cooling was significantly greater in CA-17 than it was in CA-1 and CA-16 (*P*<0.05; SP>45%) (Table 2).

**Table 2:** Body temperature, heart rate (HR), metabolic heat production (MHP), oxygen consumption (VO_2_) and cold-strain index (CSI) before and after body cooling in the CA-1, CA-16 and CA-17 sessions.

	CA-1 session			CA-16 session			CA-17 session		
	Before	After	Δ	Before	After	Δ	Before	After	Δ
T_mu_ (°C)	36.6±0.1	30.6±0.7 *	−5.9±0.8	36.4±0.1	30.6±0.4 *	−5.8±0.4	36.3±0.1	29.9±0.5 * # †	−6.4±0.5 # †
T_re_ (°C)	37.0±0.1	35.7±0.1 *	−1.4±0.1	37.0±0.1	35.52±0.02 * #	−1.5±0.1	37.0±0.1	35.1±0.1 * # †	−1.9±0.1 # †
T_sk_ (°C)	32.1±0.2	19.0±0.4 *	−13.1±0.5	32.1±0.1	18.3±0.3 * #	−13.8±0.3	32.0±0.1	19.5±0.4 * †	−12.5±0.5
HR (beats min^−1^)	65.1±2.4	84.1±5.1 *	18.9±3.9	63.8±2.9	81.0±3.0 *	17.2±3.2	62.8±2.3	78.0±2.7 *	15.2±2.4 #
MHP (W/m^2^)	38.7±3.6	162.2±8.9 *	123.5±10.2	52.3±1.3 #	167.8±15.3 *	115.5±15.1	49.1±3.0 #	145.5±7.7 * # †	96.4±7.7 # †
VO_2_ (ml kg min^−1^)	3.6±0.3	12.9±0.9 *	9.0±1.0	4.5±0.2 #	13.3±1.5 *	8.5±1.4	4.4±0.2 #	11.5±0.9 * # †	6.9±0.8 # †
CSI		5.9±0.4			7.0±0.4 #			7.9±0.5 # †	

*P<0.05, compared with before cooling;

# *P*<0.05, compared with the CA-1 session;

† *P*<0.05 – compared with the CA-16 session.

Δ  =  mean difference between before and after cold exposure. Values are means ± SEM.

### The effect of CA on body temperature gradient

**Figure 5:**
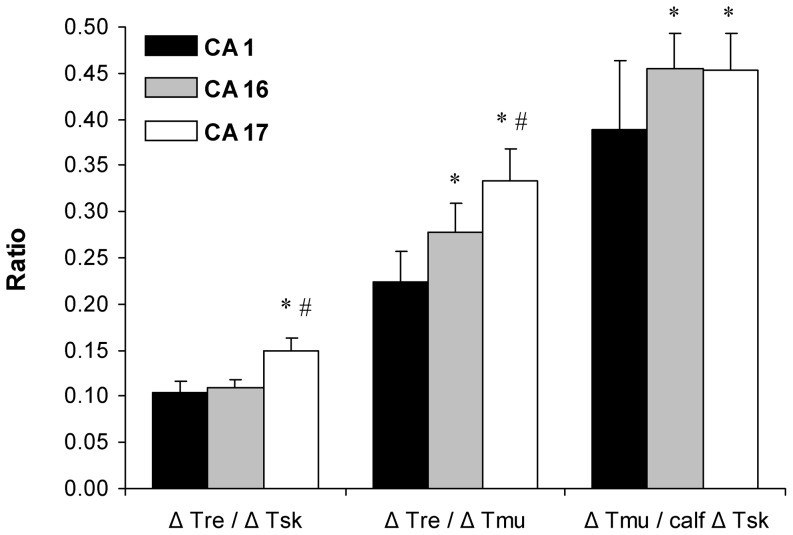
Body temperature gradient change before (CA-1 session) and after (CA-16 and CA-17 sessions) cold acclimation. * *P*<0.05, compared with CA-1; # *P*<0.05, compared with CA-16. Values are means ± SEM.

In the CA-16 session, T_re_ was decreased to a greater extent in relation to T_mu_, and T_mu_ was decreased to a greater extent in relation to T_sk_ compared with the changes observed in CA-1 (*P*<0.05; SP>45%; Figure 5). The immersion of the subjects in water for the same time as that used in the CA-1 session led to a greater T_re_ decrease in relation to T_sk_ and T_mu_, and T_mu_ was decreased to a greater extent in relation to T_sk_ (*P*<0.05; SP>45%).

### Time course of HR

**Figure 6:**
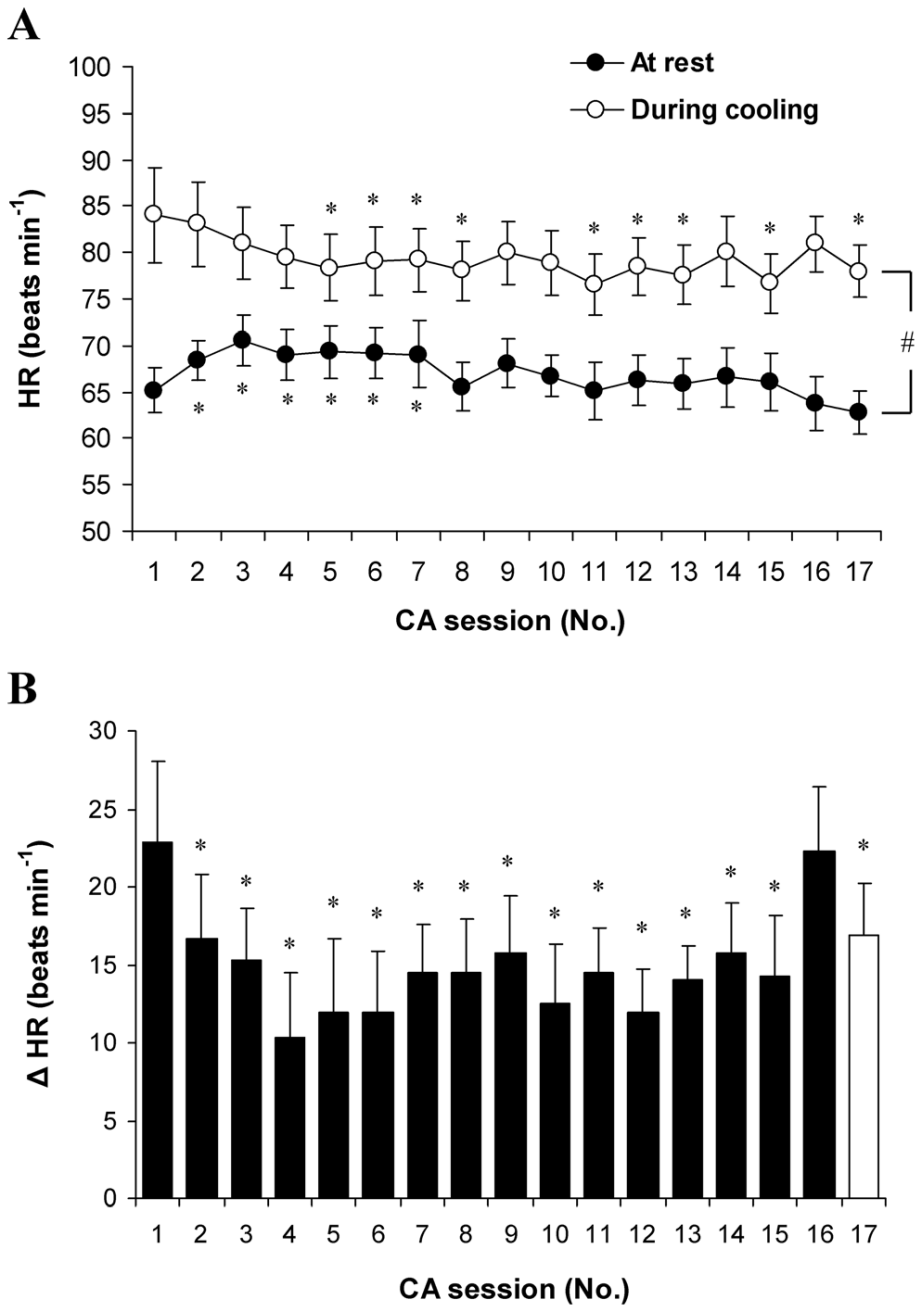
Heart rate (HR) throughout cold acclimation. HR at rest and during cooling (A), and change from at rest to during cooling (B). * *P*<0.05, compared with CA-1. Δ HR  =  mean difference between HR before and HR during cold exposure. Values are means ± SEM.

HR increased significantly during body cooling in all CA sessions (*P*<0.001; SP>99%; Figure 6A). A significantly lower mean HR during cooling was observed in the CA-5 session compared with the CA-1 session (*P*<0.05). HR before cooling increased significantly from the CA-2 to the CA-7 sessions (*P*<0.05; SP>45%) and then returned to the pre-acclimation level. HR before compared with during cooling decreased significantly from the CA-2 to the CA-15 and CA-17 sessions (*P*<0.05; SP>45%; Figure 6B). No difference between the CA-1 and CA-16 sessions was observed (*P*>0.05).

### Time course of VO_2_, MHP, cold shock and subjective indicators of cold stress

**Figure 7:**
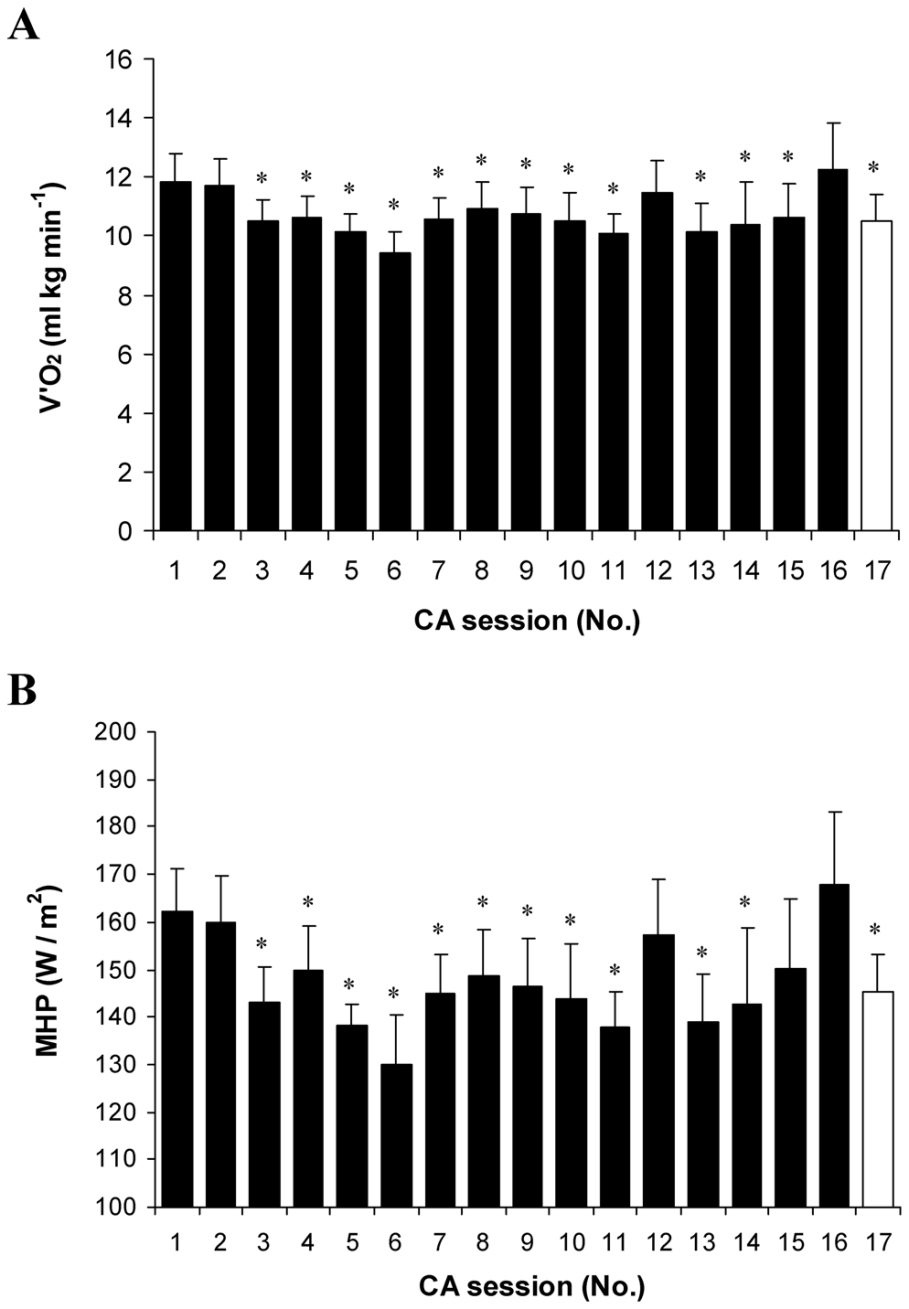
VO_2_ and metabolic heat production (MHP) throughout cold acclimation. VO_2_ (A) and MHP (B). * *P*<0.05, compared with CA-1. Values are means ± SEM.

**Figure 8:**
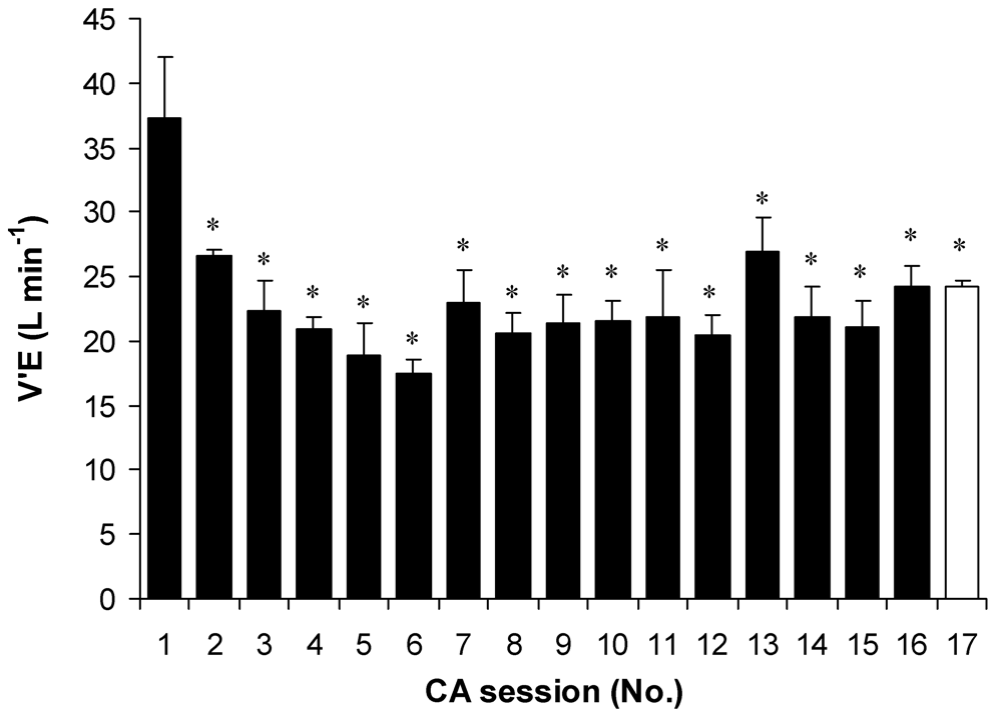
Hyperventilation throughout cold acclimation. * *P*<0.05, compared with CA-1. Values are means ± SEM.

**Figure 9:**
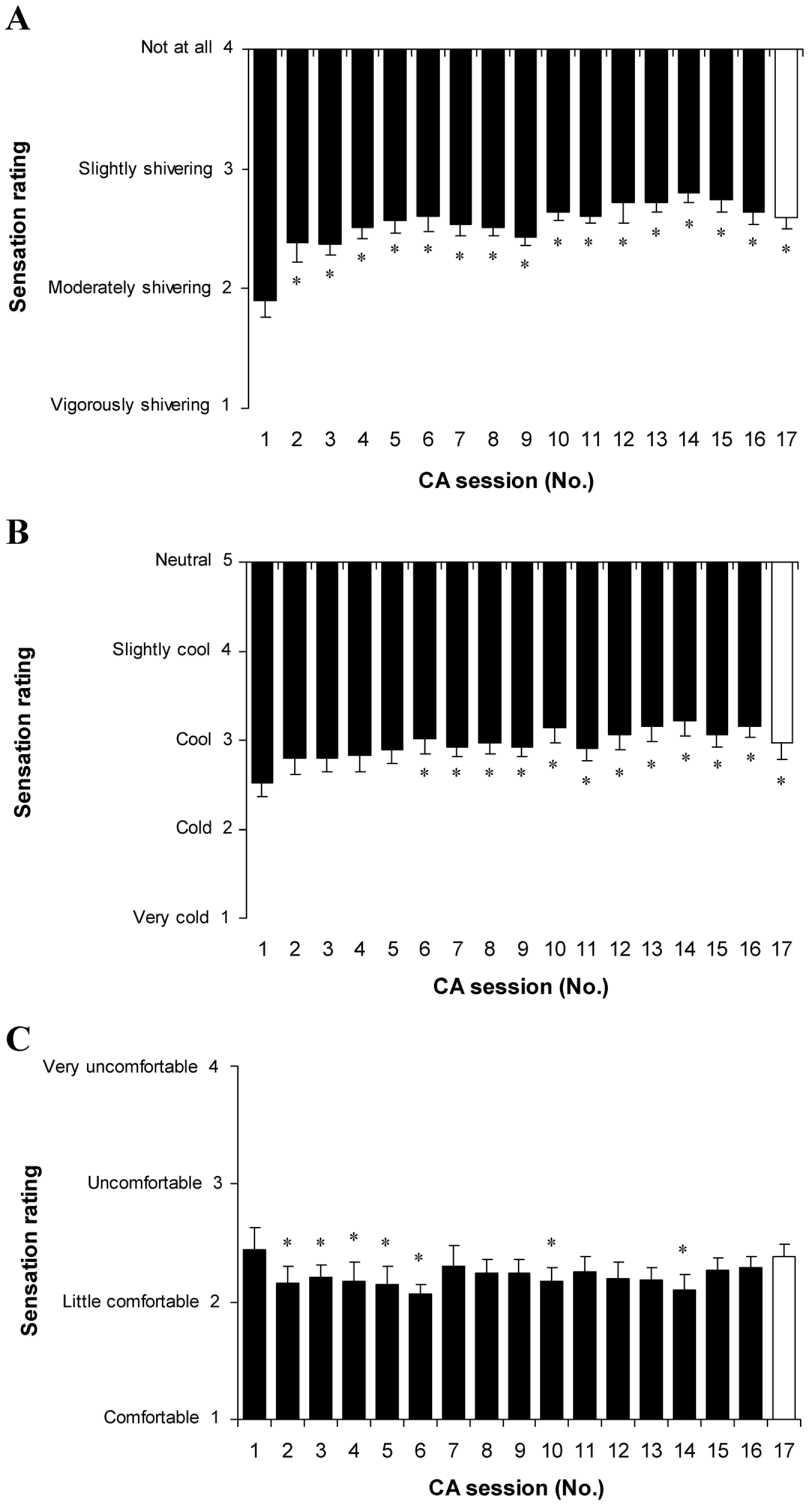
Shivering, thermal and comfort sensation throughout cold acclimation. Shivering (A), thermal (B) and comfort (C) sensation. * *P*<0.05, compared with CA-1. Values are means ± SEM.

Throughout the CA protocol, VO_2_, MHP and cold shock values decreased consistently in the first six CA sessions, and reached their lowest value at the CA-6 session (*P*<0.05, SP>80%; compared with CA-1; Figures 7 and 8), and then increased significantly (*P*<0.05; SP>45%; comparison of CA-6 with CA-7 to CA-17). VO_2_ and MHP returned to pre-acclimation levels (*P*>0.05; comparison of CA-1 with CA-16). Immersion of the subjects in water for the same time as that used in the CA-1 session lowered VO_2_, MHP and cold shock (*P*<0.05; SP>45%; comparison of CA-1 with CA-17). Notably, subjective shivering sensation decreased consistently and reached its lowest value at the CA-14 session (*P*<0.05; compared with CA-1), and then did not change until the end of the CA protocol (*P*>0.05; comparison of CA-14 with CA-16 and CA-17; Figure 9A). The CA protocol yielded a significant reduction in thermal and comfort sensations (*P*<0.05; Figure 9B, C).

### The effect of CA on CSI and blood variables

The CSI after body cooling was 5.9±0.4, 7.0±0.4 and 7.9±0.5 in the CA-1, CA-16 and CA-17 sessions, respectively (*P*<0.05; SP>80%; comparison between sessions) (Table 2). In the CA-1 session, head-out cooling yielded significant changes in all blood variables measured (*P*<0.05; SP>80%; comparison of pre- with post-cooling) (Table 3). The concentrations of cortisol and epinephrine, leucocyte count and the percentage of neutrophils, lymphocytes and monocytes did not change significantly between before and after body cooling in CA-16 and CA-17 (*P*>0.05). The increase in plasma norepinephrine level from before to after body cooling was significantly greater in the CA-1 than it was in the CA-16 and CA-17 sessions (*P*<0.05; SP>60%).

**Table 3:** Blood variables before and after body cooling in CA-1, CA-16 and CA-17 sessions.

	CA-1 session			CA-16 session			CA-17 session		
	Before	After	Δ	Before	After	Δ	Before	After	Δ
Norepinephrine, ng/ml	6.7±1.4	41.1±7.9 *	34.3±8.8	9.3±0.9 #	36.0±6.7 *	26.7±6.0 #	9.2±0.7 #	31.0±6.7 *	21.8±6.2 #
Epinephrine, ng/ml	1.6±0.4	4.6±1.1 *	3.0±1.2	2.9±1.0	2.9±1.1	−0.1±1.7 #	2.8±1.0	2.4±0.7	−0.5±1.6 #
Cortisol, nmol/l	587.2±27.4	622.3±22.3 *	34.3±14.5	546.6±29.3 #	525.2±25.1 #	−21.3±15.5 #	539.2±29.3 #	543.4±33.4 #	4.2±2.3 #
Leucocytes, ^×^10^9^/L	6.2±0.5	11.1±1.6 *	4.8±1.4	6.4±0.6	7.5±0.9	1.1±0.3 #	6.3±0.4	8.0±1.3	1.7±1.4 #
Neutrophils, %	50.8±4.6	72.0±5.3 *	21.1±1.7	52.1±4.3	61.9±3.5	9.8±1.9 #	51.6±4.4	62.1±4.7	10.6±1.5 #
Lymphocytes, %	36.9±4.5	19.8±4.5 *	−17.1±1.6	35.4±4.3	28.1±3.1	−7.3±2.0 #	36.1±4.4	27.4±3.5	−8.7±1.2 #
Monocytes, %	9.1±0.5	7.2±1.1 *	−1.9±1.0	9.7±0.6	8.6±0.6	−1.1±0.3 #	9.5±0.6	8.7±1.0	−0.8±0.2 #

*P<0.05, compared with before cooling;

# *P*<0.05, compared with the CA-1 session.

Δ  =  mean difference between before and after cold exposure. Values are means ± SEM.

### Correlation between markers of cold stress

We found no significant correlations (*r*<0.5) between BMI and changes in body temperature, CSI, subjective sensations, stress markers (concentration of cortisol, epinephrine and norepinephrine) or immune variables (leucocytes, neutrophils, lymphocytes and monocytes). A significant strong inverse correlation was found between BMI and ΔMHP in the CA-1, CA-16 and CA-17 sessions (*r* = −0.69, *r* = −0.89 and *r* = −0.89, respectively; *P*<0.05).

## Discussion

### Time course of CA markers and strategies

This study demonstrated that a repeated 17-session head-out cold-water immersion over 20 days induced CA. In the first experimental condition, under which T_re_ decreased to the set point of 35.5°C and/or the 170 min cooling time elapsed, a significantly greater decrease in T_sk_ and T_re_ after cooling and no change in MHP during cooling were found in the CA-16 session compared with the CA-1 session. These are changes in physiological responses that are somewhat characteristic of the hypothermic–insulative type of acclimation [29], and were consistent with other findings [56], [22]. However, the novel observation that, in the second experimental condition (CA-17, in which the subjects were immersed in water for the same time as that used in the CA-1 session), the CA was of the hypothermic type (which was indicated by a reduction in MHP) and resulted in a greater decrease (∼0.6°C) in T_re_ and no change in T_sk_.

It has been shown that, in cold-adapted winter swimmers, the thermoregulatory threshold for induction of cold thermogenesis was 0.34°C (T_re_) lower than that observed in non-adapted individuals [53]. In the CA-17 session, the subjects were immersed in cold water for about 25 min longer than they were in the CA-16 session. The rate of T_re_ decrease did not differ between those two sessions (Figure 3). However, T_mu_ and T_re_ were decreased to a greater extent (about 0.41°C and 0.65°C in CA-17, and about 0.02°C and 0.17°C in the CA-16 compared with the CA-1, respectively). A possible explanation for the shift from hypothermic–insulative (indicated in the CA-16 session) to hypothermic (indicated in CA-17) types of acclimation after CA might involve the thermoregulatory threshold, which was affected (induced) for cold thermogenesis by a greater decrease in T_mu_ and T_re_ in the CA-17 session, even when compared with winter swimmers.

Our study was the first to measure limb intramuscular temperature after CA. During cold-water immersion, the greater change and decrease in T_re_ in relation to changes in T_mu_ and T_sk_ was found in the CA-17 (compared with the CA-1) (Figure 6), which might indicate a redistribution of body heat stores from the core areas to total shell insulation (i.e., muscle and superficial shell). However, in both experimental conditions (CA-16 and CA-17), T_mu_ was decreased unexpectedly to a greater extent in relation to calf T_sk_ compared with that observed in CA-1. This might indicate a reduction in limb insulation, which results in greater heat loss from muscles to the skin, and then to the environment. This finding contrasts with the observation of Young *et al.* [56] that the maintenance of a warmer muscle shell after CA occurred at the expense of a cooler superficial shell.

There is recent evidence that non-shivering thermogenesis (metabolic response strategy) by the sympathetic system is induced by norepinephrine. A component of this metabolic response in healthy men and body fat content (i.e., BMI and/or body fat percentage) correlate inversely with BAT content [52], [35]. Our finding of a significant negative correlation between BMI and changes in MHP before and after acclimation is consistent with this inverse relationship between body fat content and BAT. The stronger inverse correlation (*r* = −0.69 *vs. r* = −0.89 and −0.89 in the CA-1, CA-16 and CA-17 sessions, respectively) found in this study after CA suggests indirectly the presence of increased mitochondrial heat production in more activated BAT. This correlation identified in our study is in line with the recent study performed by van der Lans *et al.* [51], in which the administration of a 10-day CA protocol in humans increased BAT presence and activity either before or after CA.

In the current study, the time course of CA (which we believe was studied here for the first time) revealed a significantly greater decrease in MHP, VO_2_, shivering sensation and T_re_, and no change in T_sk_ during the first six CA sessions. This indicates the development of a hypothermic type of acclimation. Interestingly, under such an acclimation pattern (i.e., more rapid and greater decrease in T_re_), the subjects felt less discomfort and cold sensation during cold-water immersion. From CA-6 to CA-16, a further decrease in shivering, increase in MHP and VO_2_, no change in T_re_ and decrease in T_sk_ indicate a shift toward an insulative–metabolic type of acclimation. A further decrease in psychological cold sensation was also observed. Taken together, these data allow us to speculate only that sessions CA-6 to CA-7 in our study might indicate the threshold point for the thermoregulatory shift from shivering-derived thermogenesis to brown-fat-derived (i.e., non-shivering) thermogenesis. Notably, this shift in thermogenesis fit well (i.e., at the point of about 1 week of CA non-shivering-derived thermogenesis predominates proportionally in relation to shivering-derived thermogenesis) with the principal sketch of classical non-shivering thermogenesis introduced by Cannon, Nedergaard [10] based on an experimental animal (mouse) model that had been acclimated to cold.

Although in this study we did not measure day-by-day MHP and VO_2_ under thermoneutral conditions, the observations that, in the first part of the CA, there was an increase in T_re_ (Figure 2C) and HR (Figure 5A) and no change in T_sk_ (Figure 4A) were novel. Moreover, in the CA-16 and CA-17 sessions, at thermal neutrality, MHP and VO_2_ increased significantly (Table 2) and no changes in resting T_re_, T_sk_ and HR were found compared with pre-acclimation values. However, this might indicate the presence of a metabolic type of thermogenesis. We have not found any data in the literature regarding how day-by-day repeated cold stress influences the time course of physiological markers under thermoneutral conditions. Nevertheless, our findings are in conflict with other similar research data that showed no change in MHP and a decrease in T_re_ and T_sk_ at thermal neutrality after CA, and was denoted as classical hypothermic type of adaptation [56], [5], [22], [53]. The main explanation for this discrepancy is that, here, we used a more intensive CA protocol (i.e., 17 head-out cold-water immersions (14°C) over 20 days until a T_re_ 35.5°C was reached or the time of 170 min had elapsed). Moreover, we did not re-warmed the subject's right after the end of the cold-water immersion session, which caused a further decrease of about 0.2–0.3°C in T_re_ (a total decrease in T_re_ per session of about 1.5–1.8°C was observed) and a slower return to thermoneutral level. This implies that the cooling effect lasted for a few additional hours, until it was compensated or super-compensated fully by MHP resources.

It has been shown that the stimulation of cutaneous cold receptors upon sudden and/or initial immersion in cold water (<25°C) initiates uncontrollable hyperventilation, tachycardia and a reduced breath-holding time [50], [29]. These responses are thought to be precursors to drowning; therefore, the majority of accidental deaths occur in open water [50]. In our study, the initial head-out water immersion (first minute of immersion in the CA-1 session) at 14°C resulted in a 7-fold increase in minute ventilation. This so-called “cold-shock” response was already reduced in the CA-2 session (by about 34%), and reached its lowest value (of about 53%) at the CA-6 session. Our results are in agreement with those of Golden, Tipton [16], who found a similar day-by-day decrease in ventilatory volume during the first minute of head-out resting immersion in water at 15°C over a 7-day acclimation period. Those authors suggested that the ventilatory response might have a longer time course of adaptation, as V′E was still 2.5 times higher than that observed in thermoneutral immersion. In contrast with their observation, in our study, hyperventilation increased significantly (by about 24%) in CA-7 compared with CA-6, and then did not change until the end of the CA protocol. The precise mechanisms underlying the changes in hyperventilation observed throughout the CA are not well understood, but might involve alterations in the spinal cord or the higher centres of the CNS [16], [29].

**Figure 10:**
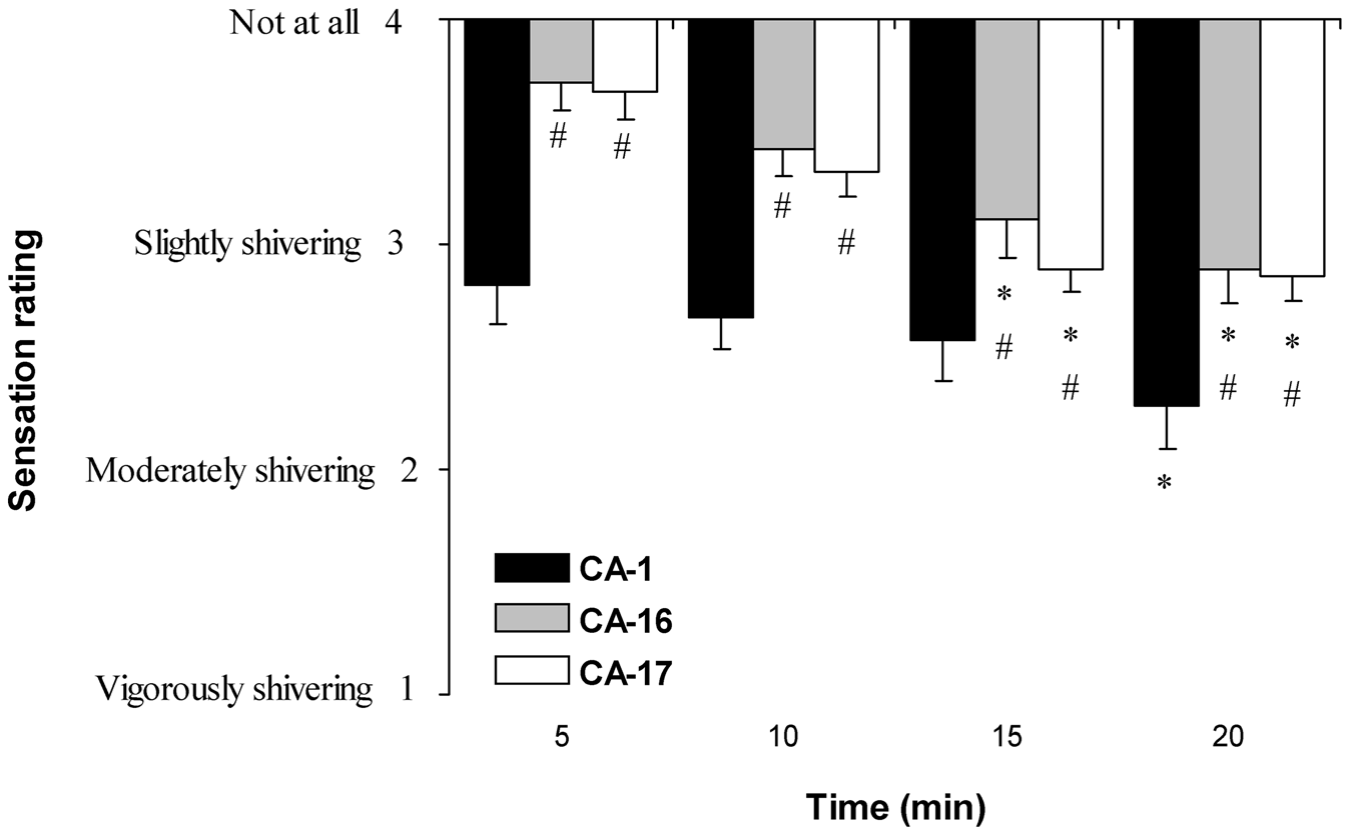
Shivering sensation during the first 20(CA-1) and after cold acclimation (CA-16 and CA-17). * *P*<0.05, compared with a 5 min; # *P*<0.05, compared with the CA-1 session; Values are means ± SEM.

It has been shown that, in the initial phase of cold-water immersion, cold thermogenesis in non-adapted subjects is independent of changes in T_re_, which indicates that cold thermogenesis is mainly induced by changes in peripheral (i.e., skin thermoreceptors) temperature input [5], [53]. The shift from peripheral to central temperature input-based thermoregulation was indicated in the late phase of cooling (i.e., after the first 10 min of cooling) by the observation of an inverse relationship between heat production and T_re_. In cold-adapted winter swimmers, this shift disappears during cold-water immersion; cold thermogenesis is solely related to changes in T_re_, indicating that the central temperature input predominates in the activation of the heat-production mechanism [5], [53]. In our study, blunted cold-shock and HR response, delayed onset and reduced intensity of shivering (Figure 10) and a more rapid and greater decrease in T_re_ compared with changes in T_sk_ were in agreement with the findings of several other studies [22], [11], [53], [46] and are indicative of an adaptive shift after CA from peripheral-to-central to central temperature input thermoregulation of hypothalamic thermoregulatory control centres by modifying sensory functions [5], [53].

### The effect of CA on neuroendocrine and immune-function activity

The results on the effects of CA on catecholamine levels are inconsistent. For instance, Young *et al.* [56] showed that 24 head-out cold-water (18°C) immersions (for 90 min) over 5 weeks yielded a significantly greater increase in norepinephrine and no change in epinephrine concentration during a cold-air test. In contrast, Radomski, Boutelier [39] observed that subjects who had completed pre-adaptation procedures (i.e., nine head-out cold-water (15°C) immersions (for 20–60 min) over 2 weeks exhibited a decrease in overnight urinary norepinephrine and an increase in epinephrine excretion after a 20-day Arctic sojourn. Surprisingly, however, we failed to find any other study investigating the effect of a repeated cold-water immersion protocol on catecholamine levels during a cold-water test. In principle, cold-water immersion results in greater and more rapid lowering of body temperature than does cold-air exposure [56], and cold-water immersion might cause greater CSI than does cold air exposure. More confusing data were reported by the study performed by Vybiral *et al.* [53], who did not find any differences in catecholamine levels between winter swimmers and non-adapted subjects during a 60 min head-out cold-water (13°C) immersion test. It is possible that the main reason for the inconsistencies observed regarding catecholamine levels between studies is the small size of the samples (≤6), different acclimation protocol and insufficient cold stress applied for testing. In our study, at thermal neutrality, there was a non-significant increase in the concentration of norepinephrine and epinephrine, which might in part explain the increase in MHP at thermoneutrality after CA. Moreover, our CA protocol caused a blunted effect on plasma catecholamine release during cold-water immersion, and the changes in catecholamine release did not depend on exposure time (CA-16 *vs.* CA-17). Lesna *et al*. [27] showed that, in humans, epinephrine rather than norepinephrine thermogenesis is potentiated by cold adaptation. In their study, epinephrine was administered to winter swimmers and caused a greater increase in MHP, whereas the administration of norepinephrine had no effect compared with that observed in non-adapted individuals. However, well-established evidence indicates that BAT in humans is activated by elevated plasma catecholamine levels [45], [54]. Evidence from an animal model revealed that, after CA, the heat production in thermoneutrality from BAT ceases, but the tissue remains recruited [10]. An injection of norepinephrine elicits a much greater response in MHP compared with that observed before CA, and this increase is fully due to increased BAT-derived thermogenesis. The absence of changes in epinephrine and the significant, but blunted, increase in norepinephrine found in our study suggest that heat production after CA is compensated more by norepinephrine-induced non-shivering thermogenesis than by epinephrine-induced shivering thermogenesis. Another alternative explanation is that the adrenal medulla became fatigued because of the intensive CA protocol [11].

Despite the fact that CSI increased significantly after acclimation, a cold-stress stimulus under both experimental conditions (CA-16; CA-17) had a blunted effect on the activation of the sympathetic–adrenomedullary (catecholamine release) and hypothalamic–pituitary–adrenocortical (cortisol release) systems. The blunted release of cold-induced stress markers after CA might be one of the main reasons why CA results in blunted activation of innate response and blunted suppression of the specific immune system. This indicates immune-system adaptation. We believe that longer and more detailed studies are needed to explain fully whether (and to what extent) acclimation to acute severe-cold exposure stimulates or suppresses the immune response.

## Conclusion

As expected, the data obtained in this study suggest that the subjects exhibited a thermoregulatory shift from peripheral-to-central to central input thermoregulation, as well as from shivering to non-shivering thermogenesis throughout the CA. Contrary to our expectation, in the first six CA sessions, a hypothermic type of acclimation was found; further CA (CA-7 to CA-16) led to a transitional shift to a hypothermic–insulative type of acclimation. Interestingly, when the subjects were immersed in water for the same time as that used in the CA-1 session (CA-17), the CA led to a hypothermic type of acclimation. Finally, the presence of a metabolic type of thermogenesis was evident only under thermoneutral conditions. Despite the fact that CSI was increased significantly after acclimation, cold-water immersion decreased the concentration of cold-stress markers (cortisol, epinephrine and norepinephrine), reduced the activity of the innate immune system, suppressed specific immunity to a lesser degree and yielded less discomfort and cold sensation. As expected, we found a negative correlation between BMI and ΔMHP before and after acclimation, which is consistent with the inverse relationship detected between body fat content and BAT [52], [35].
